# Diet-Induced Rabbit Models for the Study of Metabolic Syndrome

**DOI:** 10.3390/ani9070463

**Published:** 2019-07-20

**Authors:** Wilson M. Lozano, Oscar J. Arias-Mutis, Conrado J. Calvo, Francisco J. Chorro, Manuel Zarzoso

**Affiliations:** 1 Department of Physiology, Universitat de València, 46010 Valencia, Spain; 2 CIBERCV, Instituto de Salud Carlos III, 28029 Madrid, Spain; 3 Department of Cardiology, Hospital Clínico Universitario, 46010 Valencia, Spain; 4 Department of Physiotherapy, Universitat de València, 46010 Valencia, Spain

**Keywords:** metabolic syndrome, animal models, rabbit, dietary regimes

## Abstract

**Simple Summary:**

In recent years, obesity and metabolic syndrome (MetS) have become more prevalent owing to increased unhealthy habits and sedentary lifestyles, becoming public health problems. Experimental studies have allowed us to explore the mechanisms underlying the pathophysiological processes related to MetS. Several research protocols can be carried out with minimal staff, maintenance, and resources in animals such as rabbits. High-fat diets enriched with other components, mainly cholesterol and sugars, result in the rapid development of hypercholesterolemia and vascular alterations as a response to dietary manipulation. Furthermore, other experimental models, including transgenic rabbits with altered expression of specific genes, have been used to decrease the duration of experimental studies and increase the response to diet.

**Abstract:**

Obesity and metabolic syndrome (MetS) have become a growing problem for public health and clinical practice, given their increased prevalence due to the rise of sedentary lifestyles and excessive caloric intake from processed food rich in fat and sugar. There are several definitions of MetS, but most of them describe it as a cluster of cardiovascular and metabolic alterations such as abdominal obesity, reduced high-density lipoprotein (HDL) and elevated low-density lipoprotein (LDL) cholesterol, elevated triglycerides, glucose intolerance, and hypertension. Diagnosis requires three out of these five criteria to be present. Despite the increasing prevalence of MetS, the understanding of its pathophysiology and relationship with disease is still limited. Indeed, the pathological consequences of MetS components have been reported individually, but investigations that have studied the effect of the combination of MeS components on organ pathological remodeling are almost nonexistent. On the other hand, animal models are a powerful tool in understanding the mechanisms that underlie pathological processes such as MetS. In the first part of the review, we will briefly overview the advantages, disadvantages and pathological manifestations of MetS in porcine, canine, rodent, and rabbit diet-induced experimental models. Then, we will focus on the different dietary regimes that have been used in rabbits to induce MetS by means of high-fat, cholesterol, sucrose or fructose-enriched diets and their effects on physiological systems and organ remodeling. Finally, we will discuss the use of dietary regimes in different transgenic strains and special rabbit breeds.

## 1. Introduction

Sedentary lifestyles and excess caloric intake from a diet high in fats and sugars have made obesity and metabolic syndrome (MetS) international public health problems [1]. MetS is a condition that has been gaining interest since it was originally described by Reaven in 1988. This high-risk metabolic condition has been variably defined over time in several studies [2,3,4,5]. All definitions agree that MetS is a set of metabolic modifications, while maintaining discrepancies regarding the deficiencies that compose MetS at the time of diagnosis. The alterations include insulin resistance, excess abdominal fat, dyslipidaemia, hypertension, endothelial dysfunction, and a general pro-inflammatory status. Metabolic dysregulation in MetS leads to an enhanced risk of cardiovascular disease (CVD), increased incidence of stroke, the appearance of arrhythmic events, and sometimes, sudden cardiac death and mortality. Furthermore, it has been shown to increase the risk of type 2 diabetes onset. However, this syndrome could be minimized, or even eluded, because the associated metabolic deficiencies are related to an unhealthy lifestyle [6,7].

Due to the large increase in the worldwide prevalence of MetS and cardiovascular diseases, it is important to elucidate the mechanisms that are involved in its pathophysiology and concurrent cardiovascular consequences [8,9,10]. Proper models to study the mechanisms of progression of the disease are precisely needed to be able to extrapolate research findings to the clinical setting. Indeed, for this purpose, by using in-vivo models, researchers may obtain an essential tool to evaluate these pathophysiological changes related to obesity and MetS [11,12,13]. The selection of the animal model should allow an appropriate representation of the clinical manifestations of the human condition as far as possible [14,15,16,17,18].

**Figure 1:**
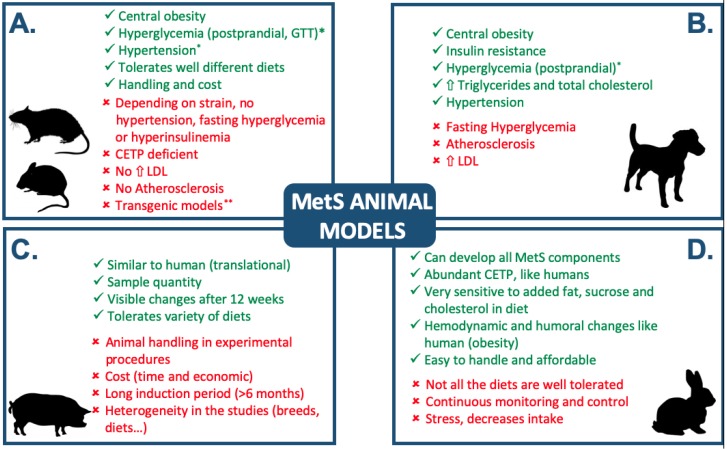
Comparation of MetS animal models. (**A**) Rats and mice; (**B**) Dogs; (**C**) Pigs; (**D**) Rabbits. “√” and “×” indicates advantage and disadvantage, respectively. CEPT: cholesteryl ester transfer protein. GTT: glucose tolerance test. Modified from Arias-Mutis et al. [16].

To date, rats, mice, dogs, pigs, and rabbits have been used as animal models, but some of them fail to exhibit all the characteristics of MetS components in humans (Figure 1) [19,20]. In rodent models, induction using diet adjustments has been widely used in studies on obesity, arterial hypertension, and MetS. Despite the ease of handling rodents, they present important drawbacks because some strains do not develop all the MetS components. Moreover, obesity depends on factors associated with the experimental feed diet, and it can manifest in the case of normal or even reduced food intake in genetically modified strains [16].

On the other hand, canine models do not show all the components of MetS because the development of endothelial damage or fasting hyperglycaemia in dogs is questionable. In addition, porcine models present high anatomical and physiological similarity with humans [18,21,22]. Therefore, they can offer significant predictive power for MetS components, but their maintenance and need for trained personnel and complex resources for experimental procedures make porcine models very laborious and expensive to use [16].

Experimentation in rabbits has succeeded because the animal represents an intermediate point between large animals and rodent models. The rabbit model can allow for several research protocols to be carried out with minimal staff, maintenance, and resources. In addition, it has been documented that rabbits fed a high-fat diet show hemodynamic and neurohumoral changes similar to those observed in obese humans [19]. As herbivorous animals, rabbits are susceptible to high-fat diets, and they have high baseline plasma lipid transfer protein (CETP) and low-density lipoprotein (LDL) profiles, similar to those in humans [23,24]. Humans as well as rabbits are LDL mammals, which, together with their similarities in lipoprotein metabolism, contributes to their usefulness as a translational model for the study of MetS and other atherosclerotic diseases [25].

The rabbit model can provide multiple advantages, owing to its similarity with human physiology and its affordability for chronic protocols and monitoring. Despite these great advantages, the rabbit has not been widely used in experimental protocols including the administration of diets rich in fat and sugar to induce MetS. Therefore, the main objective of this review is to describe the different dietary regimes in the rabbit model that allow the expression of components involved in MetS development.

## 2. Diet Regimes

Since the laboratory rabbit is a herbivore, its typical feed contains approximately 2% vegetable fat, 15% protein, 40–50% carbohydrates, and 15–25% fibre [26,27]. In New Zealand white rabbits, this feed generates typical plasma cholesterol concentrations in the range of ~30–65 mg/dL. Young animals (<3 kg of body weight) are usually at the higher end of this range. HDL is the most abundant lipoprotein in normal rabbits [26], transporting more than half of circulating cholesterol in fasting rabbit plasma [27,28]. The results are expressed rapidly with a marked increase in very low-density lipoprotein (VLDL) and LDL by supplementing the diet with fat and even cholesterol [27,29,30].

Therefore, different nutritional approaches could induce MetS in experimental rabbit models, including administering a single increased component (fat, cholesterol, sucrose, or fructose) or a combination of them [31,32,33,34]. Most researchers use strains of New Zealand white rabbits, and the duration of treatment ranges from 8 to 36 weeks. In general, they are compared to a control group that receives a standard diet that contains a fixed balance between protein, soybean fat, carbohydrates, and fibre [23,35,36,37].

In general, the experiment begins approximately 2–3 weeks after the acclimatization of the animal. Then, the intake for both groups (control and trial) is continuously controlled. This monitoring can be carried out using restricted diets with an equal number of calories per day according to the weight of the animal. Another form of diet administration is to allow “ad libitum” water intake without restriction, which is self-regulated by the same animal according to the caloric density [14,19,23,36,38].

In addition, dietary modification has been combined with genetic manipulation, so strains simulate human familial hypercholesterolemia, such as the Watanabe hereditary hyperlipidaemic rabbit (WHHL) or rabbits with ApoE gene suppression, for studies related to lipid metabolism and its consequences [39].

### 2.1. High-Fat Enriched Diet

Fats are some of the main macromolecules that are usually included in diets. Fats contain the highest caloric content (9 Kcal/g) out of all macromolecules. Fat is an ester that is usually known as a triglyceride composed of three chains of fatty acids and glycerol. Large amounts of glycerol and fatty acids are freely mobilized by the bloodstream. Fatty acids run free in plasma, and they are the major substrate for the production of VLDL and LDL in the liver [40]. This is how most free fatty acids are synthesized in the liver and adipose tissue to form new triglycerides or new reserve fat, and this metabolic event is known as lipogenesis. The rate of production is dependent on the production of glycerol-3-phosphate (glycolysis), and the release rate of fatty acids depends on adipocytes [41].

Many researchers have used different high-fat diets (Table 1) oscillating between 10% and 60% of the total energy consumed by the animal. The source of the fatty component can be very diverse: oil derived from plants (corn, coconut, safflower, peanut, linseed, palm, and olive) or fats derived from animals (cow butter and lard) [42].

Diets rich in fat have been widely used to induce obesity and MetS in experimental models. Their ability to induce obesity has been demonstrated by many studies [19,23,43,45]. However, Brunner et al. did not find significant differences in weight between control animals and high-fat diet (47.8% kcal) animals [36]. Despite their high consumption of fat, these animals did not gain weight. This is probably due to self-regulated feeding based on caloric intake, rather than bulk intake, in rabbits or because the diet was not well-tolerated; other factors may be related to the level of activity or stress [36].

Other studies indicated that a high-fat diet is effective in promoting hyperglycemia, insulin resistance, dyslipidemia, and an increase in free fatty acids in the blood, either independently or concurrently [46]. Carroll et al. showed that feeding New Zealand white female rabbits with excess calories from a diet high in fat at 15% (10% corn oil and 5% lard) induced obesity, resulting in increased blood pressure [19]. Waqar et al. reported that white Japanese male rabbits did not develop obesity despite a high-fat diet at 10% of coconut oil administration for 22 weeks. However, they did find an increase in blood pressure that was attributed to glomerular function changes due to Na^+^ retention. In addition, the group that received a high-fat diet at 3% showed adverse effects on lipid and glucose metabolism and increased blood pressure, suggesting that blood pressure is more sensitive than other alterations induced by high-fat intake without obesity [23].

On the other hand, high-fat diet studies over 12 weeks showed a significant increase in visceral adipose tissue, plasma glucose, cholesterol, triglycerides, mean arterial pressure, and a marked decrease in glucose intolerance and HDL [31,47]. These findings agree with those reported by Morelli et al. and Meneschi et al., who reported hyperglycemia, glucose intolerance, hypertension, dyslipidemia (hypercholesterolemia and hypertriglyceridemia), and obesity [48,49].

### 2.2. Diet Supplemented with Cholesterol

Rabbits are very sensitive to cholesterol administration, and they can rapidly develop severe hypercholesterolemia leading to atherosclerosis. Therefore, rabbits fed cholesterol are widely used for research related to hypercholesterolemia (Table 2). Rabbits fed a diet containing up to 2% cholesterol showed a rapid rise in plasma cholesterol, which can exceed 2000 mg/dL. This response can be exacerbated if extra saturated fat is added to the diet, increasing both the plasma cholesterol level and the extent of vascular lesions [25]. The high cholesterol diet leads to an increase in plasma β-VLDL levels, high ester content derived from the liver and intestine due to the efficient absorption of cholesterol, limited hepatic conversion of cholesterol to bile acids, and downregulated hepatic lipoprotein receptors [27].

Common atherogenic diets consist of 0.3% to 2% cholesterol and 4% to 8% fat per kilogram of weight. In fact, studies using diets with 2% added cholesterol showed high plasma levels of LDL, HDL, and triglycerides. In addition, significant changes in cardiovascular function (blood pressure and heart rate) were shown, producing a proarrhythmic state [38].

Therefore, the addition of cholesterol to the high-fat diet in rabbits is preferred to study the pathogenesis of diseases such as atherosclerosis. After administering a cholesterol-enriched diet, the animals present a clinical evolution that triggers the pathology, starting with an increase in total cholesterol, LDL, and VLDL, with a decrease in HDL [24]. However, rabbits show excessive hypercholesterolemia (greater than 2000 mg/dL) and massive accumulation of lipids in many organs, including the aorta and blood vessels, when using a diet containing more than 1% cholesterol for a period longer than a month. In that case, rabbits fed cholesterol often reproduce a model of atherosclerosis recognized as “non-physiological”, because the cholesterol concentration is too high in the plasma and causes unusual lesions in the aorta. Therefore, it is generally recommended that rabbits are fed with cholesterol from 0.3% to 0.5%, resulting in a reasonable elevation (compatible with human familial hypercholesterolemia) of plasma cholesterol with an average of 1000 mg/dL, without affecting the health of animals [25].

### 2.3. Sucrose and Fat-Enriched Diet

Sucrose, also called sugar, is a disaccharide that is extracted from cane or beet. It is composed of a molecule of fructose and a molecule of glucose [50]. By ingesting sugar, both molecules (glucose and fructose) are taken up via their specific transport mechanisms. The glucose uptake in glucose metabolism is negatively regulated by phosphofructokinase, which leads to the continuous entry of fructose into the glycolytic pathway. Excess fructose will turn into fat in the liver because fructose is a good substrate for fatty acid synthesis compared to glucose. Thus, fructose becomes one of the components that influences MetS development, which is induced by sucrose consumption the most [50].

Studies in rabbits showed that the weight of experimental rabbits increased significantly compared to that of control rabbits when fed a high-sucrose diet for 24 to 36 weeks (Table 3) [32,51]. Zhao et al. observed that the weight increase is mainly due to the marked accumulation of fat in the visceral tissue, especially in the mesentery and retroperitoneal fatty tissues, while that in the subcutaneous fatty tissue was not significant. In addition to finding central obesity in this experimental group, the animals had renal failure due to the increase in plasma glucose and increased insulin production. However, hypertriglyceridemia, hyperglycemia, and hypertension were not found in their experiment [51].

Helfenstein et al. administered a diet to New Zealand white rabbits composed of 40% sucrose and 10% lard for 24 weeks, with 0.5% cholesterol supplement during the first 12 weeks and 0.1% until the end of the study. Their rabbits exhibited hyperglycemia, hypercholesterolemia, and a marked increase in total cholesterol, triglycerides, and LDL after week 12, but insulin levels did not change over time. Despite the increased glucose and total cholesterol, the levels of liver enzymes and serum creatinine were not affected in their model [32].

Meanwhile, Yin et al. administered a diet with 37% sucrose and 10% pork lard for 6 months, showing a decrease in animal weight for those fed on the diet compared to the control group. They reported a significant increase in plasma glucose concentration after the fifth month and in the insulin level after the third month. The total plasma cholesterol level increased throughout the experiment, reaching three times the initial value by the end of the study. Similarly, triglycerides increased after the first month [34].

Arias-Mutis et al. administered a diet composed of fat (10% hydrogenated coconut oil and 5% lard) and sucrose (15%) for 28 weeks in New Zealand white rabbits, achieving expression of MetS components, such as obesity, hypertension, pre-diabetic state, dyslipidemia with low HDL content, and a high content of triglycerides and LDL, similar to those in humans. They also developed important changes related to insulin resistance and type 2 diabetes [14].

On the other hand, Liu et al. administered a high-sucrose diet (30%) for 48 weeks and observed unhealthy conditions in their rabbits. They reported a decrease in body weight and HDL level; an increase in the levels of fasting plasma glucose, total cholesterol, and LDL; and no significant changes in total cholesterol and insulin. In addition, they reported an apparent cardiac morphological alteration as a reflection of increased septum thickness, without significant changes in the ejection fraction [35].

### 2.4. Fructose and Fat-Enriched Diet

Fructose is commonly known as fruit sugar, and it is a monosaccharide similar to glucose and galactose. Fructose is often used as a flavor enhancer to make food more appetizing, but it has no biological role except that of an intermediate molecule during glucose metabolism [52]. Small amounts of fructose can produce a lower glycaemic response by replacing sucrose and starch in the diets of patients with diabetes [53].

Physiologically, a large chronic influx of fructose in the liver causes an accumulation of triglycerides and cholesterol because of its lipogenic properties, reducing insulin sensitivity, and producing insulin resistance and glucose intolerance [54,55]. In fact, the high consumption of fructose leads to massive fructose uptake by the liver, which is converted to fructose-1-phosphate, catalyzed by the enzyme phosphofructokinase in the presence of ATP [56].

Phosphofructokinase is a negative regulator of glucose metabolism, allowing fructose to continuously enter glycolysis. Fructose-1,6-bisphosphate is converted to pyruvate by glycolysis. Fructose then intervenes in several simultaneous processes: (a) a portion of fructose is converted to lactate from pyruvate; (b) another portion produces triphosphate that is easily converted to glucose or glycogen by gluconeogenesis; (c) carbons derived from fructose can be converted to fatty acids; and (d) inhibition of the hepatic lipid oxidation of very low-density lipoproteins (VLDL), triglyceride synthesis, and re-esterification of fatty acids [57].

As a result of this refinement, carbohydrates are rapidly absorbed and easily metabolized by the liver to produce glucose, glycogen, pyruvate, lactate, glycerol, and acyl-glycerol molecules. A high-sugar diet, particularly high fructose, has an important theoretical role in inducing MetS based on the effects on the cardiovascular and renal systems in diverse populations [58].

A study on WHHL rabbits fed a 30% fructose and 10% coconut oil diet did not show changes in body weight between groups. However, there were changes in the plasma levels of total cholesterol and triglycerides (Table 4), without alterations in HDL levels. These modifications were attributed to an increase in apolipoprotein (apo-B) and hepatic lipoproteins, VLDL and LDL [33]. Despite high free fatty acid plasma levels, plasma glucose and insulin levels did not change, and animals exhibited a delayed ability to eliminate glucose from the circulation as well as insulin resistance [33].

## 3. Other Experimental Models

Wild rabbits are sensitive to a cholesterol-enriched diet, and they rapidly develop hypercholesterolemia and atherosclerosis. Despite this, a mutation in the “Wanatabe heritable hyperlipidemic” (WHHL) Japanese White rabbit spontaneously shows hypercholesterolemia and atherosclerosis because of a genetic LDL receptor function deficiency; this animal has been used experimentally as a hereditary hypercholesterolemia model [39]. Unlike other animals, this rabbit naturally has deficient hepatic lipase activity; rabbits show 10% activity compared to animals such as rats [59]. The hepatic lipase level in WHHL rabbits is up to 40% less than that in normal rabbits [27]. Hepatic lipase plays an important role in lipoprotein metabolism, and it converts intermediate-density lipoprotein (IDL) to LDL, remodelling the long chain rich in HDL2 to a small denser HDL3 and transferring HDL to cholesterol in the liver to contribute to reverse cholesterol transport [60].

Fan et al. reported the generation of white transgenic New Zealand rabbits to identify human hepatic lipase overexpression after heparin administration. They found levels similar to those in humans and a decrease in the triglyceride and total cholesterol levels. This decrease was related to a decrease in HDL of more than five times, reaching values closer to the absence of this lipoprotein. They concluded that this outcome is a product of hepatic lipase activity on the surface of the HDL cholesterol chain, facilitating cholesterol passage in cell membranes [28].

On the other hand, Koike et al. detected overexpression of the lipoprotein-lipase transgene, with an increase in muscle and adipose tissue concentrations in transgenic WHHL rabbits. The expression of the transgene turned out to be higher than that in normal animals. In addition, they reported a marked decrease in total cholesterol and triglyceride levels, with greater changes related to decreases in VLDL and IDL, a slight increase in HDL, and a significant increase in LDL [61].

The lipoprotein lipase activity did not show changes in glucose and insulin levels, except in the subcutaneous and visceral adipose tissue, where a significant decrease was found in quantity and weight associated with the overexpression of lipoprotein lipase. In the presence of lipoprotein lipase overexpression in Watanabe rabbits, increase in cholesterol and triglycerides can be corrected, reducing their accumulation in the adipose tissue [61]. Likewise, Liu et al. demonstrated that lipoprotein lipase overexpression significantly reduces triglyceride and total cholesterol levels in normal rabbits, without significant changes in glucose and insulin levels and with an increase in insulin sensitivity [62].

Another experimental model useful in cardiovascular research is the generation of rabbits with gene suppression, or “knockouts” (KO), with recent technological innovations in gene editing such as zinc finger nuclease (ZFN), activating transcription activating nucleases (TALENs), and RNA-guided CRISPR-associated protein 9 (Cas9) [63]. Gene editing makes it possible to generate KO animals without necessarily using homologs, based on recombination and genomic manipulation, such as endothelial stem cells, which were used for several years in experimental models using mice [39].

In this regard, Niimi et al. compared the lipoprotein profiles of apoE KO rabbits to those of WHHL rabbits, demonstrating that KO rabbits are a useful model to study human hyperlipidemia. They showed a significant increase in triglycerides and total cholesterol, with the increase being six times higher than that in normal rabbits and very similar to the levels found in WHHL, after administering a diet containing 0.3% cholesterol and 3% soybean oil for 2 weeks. This article became the first to characterize a rabbit apoE KO with abnormal lipoprotein levels, and it positions the apoE KO rabbit as a useful model to study human hyperlipidemia [39].

## 4. Discussion and Conclusions

The different dietary regimes used to induce MetS in experimental rabbit models allow researchers to establish parameters in order to select the most consistent and reliable method to study hemodynamic, neurohumoral and structural changes related to MetS development, cardiovascular consequences and physiopathological similarities with humans.

The duration of the diet administration is one point of disagreement between authors. Researchers using the same dietary components vary the durations of high-fat diets from 12 to 24 weeks [19,24]. Studies on high-sucrose diets used durations of 24 to 48 weeks [32,35]. Both cases led to the development of some MetS components, but there were differences in metabolic alteration severity that could be related to the duration of the diet feed and diet composition.

However, less time is required (8 to 12 weeks) to administer high-fat diets supplemented with cholesterol [38,49]. This is due to the greater sensitivity of rabbits to cholesterol and the rapid development of biochemical and vascular changes. Therefore, it is an ideal model to study atherosclerosis using the combination of fat and cholesterol for a few weeks.

There is little evidence for the treatment of fat combined with fructose in rabbits. The only relevant study noted increases in blood plasma biochemistry, insulin resistance, decreased insulin sensitivity, and no changes in body weight 16 weeks after administering the diet in WHHL transgenic rabbits [33]. Therefore, it is necessary to discern whether cholesterol elevation is influenced by diet or if it is natural spontaneous hypercholesterolemia in WHHL rabbits [39]. In mice fed fructose, a higher efficiency was found compared to mice fed with glucose or starch, which increased the weight before the chronic intake of fructose. In addition, they developed hyperlipidemia, hypertriglyceridemia, hypertension, and glucose intolerance, which verified the development of MetS [64].

It is necessary to standardize the adequate diet administration duration, according to the treatment and the evaluation of the expected changes in the experimental series, without risking the health of the rabbits, to optimize the methodology to be used. This last aspect is important when comparing the physiological states of the human and the animal model, given that, for example, diets with a high concentration of cholesterol can generate extremely high values that cannot be compared to pathological values in humans, putting the animals’ health at risk [25]. In addition, it would be necessary to determine the daily intake to monitor the animals and assess the degree of diet tolerance, but practically none of the analyzed studies did this.

All the dietary regimens described here generate disorders in glucose and lipid metabolism. Diets supplemented with 10% fat cause insulin resistance and increase blood pressure without weight gain [23]. Diets that use 15% fat cause obesity, alter renal function, and increase predisposition to hypertension development [19]. On the other hand, treatments enriched in cholesterol increase susceptibility to systemic inflammatory status (leucocytosis and high levels of C-reactive protein) and increase plasma glucose, cholesterol, obesity, and vascular lesions such as atherosclerosis [25]. Likewise, the fat diets enriched with sugars (sucrose and fructose) show increases in all the MetS components, including hyperglycemia, hypertension, weight gain, fat accumulation (predominantly visceral) [51], total cholesterol, triglycerides [32], VLDL, and LDL [33].

Finally, it is important to include new experimental tools such as transgenic rabbits to modify individual genes that cause overexpression of lipoproteins or other effects. These models can provide other means to clarify the physiological and molecular mechanisms involved in the development of obesity, MetS, and cardiovascular disease [9,25,27]. This will elucidate MetS component expression based on the composition of the diet, the choice of animals and the duration of the diet, allowing for future understanding related to experimental MetS models in rabbits.

## Figures and Tables

**Table 1:** Effects of high-fat enriched diets on the development of MetS (metabolic syndrome).

Author	Diet	Duration	Rabbit Breed	MetS Components				
				Ob	HG	HT	DL	FFA
Waqar et al. [23]	3% coconut oil	22 weeks	Japanese White male	🗴	✓	✓	✓	✓
	10% coconut oil			✓	✓	✓	✓	✓
Brunner et al. [36]	17% fat (added palmitic, oleic and linoleic acid)	28 weeks	New Zealand White	-	-	-	-	-
Carroll et al. [19]	15% fat, 10% corn oil and 5% lard	12 weeks	New Zealand White female	✓	✓	✓	✓	✓
Alarcon et al. [43]	18% fat, 10% corn oil and 8% lard	6 weeks	Hybrid Flanders	🗴	✓	✓	✓	✓
Cervera et al. [44]	8.5% animal fat	12 weeks	Crossbred	✓	-	-	-	-
	2.5% soya full fat			✓	-	-	-	-

The symbol “✓” indicates the presence and “🗴” indicates the absence of the MetS components, while “-” indicates that the component was not evaluated in the study. Ob: obesity, HG: hyperglycemia; HT: hypertension, DL: dyslipidemia and FFA: free fatty acids.

**Table 2:** Effects of diets supplemented with cholesterol on the development of MetS (metabolic syndrome).

Author	Diet	Duration	Rabbit Breed	MetS Components				
				Ob	HG	HT	DP	FFA
Drimba et al. [38]	1.5% cholesterol and 2.6% fat	8 weeks	New Zealand White male	-	-	✓	✓	✓
Filippi et al. [31]	0.5% cholesterol and 4% peanut oil	12 weeks		✓	✓	✓	✓	✓
Maneschi et al. [49]	0.5% cholesterol and 4% peanut oil			✓	✓	✓	✓	✓
Marchiani et al. [47]	0.5 cholesterol and 4% peanut oil			✓	✓	✓	✓	✓
Morelli et al. [48]	0.5% cholesterol and 4% peanut oil			✓	✓	✓	✓	✓

The symbol “✓” indicates presence, and “-” indicates that the component was not evaluated in the study. Ob: obesity, HG: hyperglycemia; HT: hypertension, DL: dyslipidemia and FFA: free fatty acids.

**Table 3:** Effects of sucrose and fat-enriched diets on the development of MetS (metabolic syndrome).

Author	Diet	Duration	Rabbit Breed	MetS Components				
				Ob	HG	HT	DP	FFA
Arias-Mutis et al. [14]	10% hydrogenated coconut oil, 5% pork fat, 15% sucrose dissolved in water	28 weeks	New Zealand White male	✓	✓	✓	✓	✓
Helfenstein et al. [32]	10% lard, 40% sucrose and cholesterol (0.5% for the first 12 weeks and 0.1% for up to 24 weeks)	24 weeks		✓	✓	-	✓	✓
Liu et al. [35]	30% sucrose and 10% fat	48 weeks		✓	✓	-	✓	✓
Yin et al. [34]	10% pork fat and 37% sucrose	6.5 months (28 weeks)		✓	✓	-	✓	✓
Zhao et al. [51]	10% pork fat and 30% sucrose (11% protein, 11.2% fat, 10.1% fiber, 6.8% ash)	36 weeks	Japanese White male	✓	✓	✓	✓	✓

The symbol “✓” indicates presence, and “-” indicates that the component was not evaluated in the study. Ob: obesity, HG: hyperglycemia; HT: hypertension, DL: dyslipidemia and FFA: free fatty acids.

**Table 4:** Effects of fructose and fat-enriched diets on the development of MetS.

Author	Diet	Duration	Rabbit Breed	MetS Components				
				Ob	HG	HT	DP	FFA
Ning et al. (33)	30% fructose and 10% coconut oil (91% saturated fatty acids)	16 weeks	WHHL	✓	✓	🗴	✓	✓

The symbol “✓” indicates the presence and “🗴” indicates the absence of the MetS components in the study. WHHL: Watanabe heritable hyperlipidemic rabbit, Ob: obesity, HG: hyperglycemia; HT: hypertension, DL: dyslipidemia and FFA: free fatty acids.
